# Associations between pain, self-efficacy, sleep duration, and symptoms of depression in adolescents: a cross-sectional survey

**DOI:** 10.1186/s12889-021-11680-1

**Published:** 2021-09-05

**Authors:** Kristin Haraldstad, Tonje Holte Stea

**Affiliations:** 1 University of Agder, Faculty of Health- and Sport Sciences, P.O box 422, 4604 Kristiansand, Norway; 2 Department of Child and Adolescence Mental Health, Sørlandet Hospital, Kristiansand, Norway

**Keywords:** Adolescents, Pain, Self-efficacy, Sleep, Depression

## Abstract

**Background:**

Although pain has been identified as an important public health problem among adolescents, few studies have investigated possible protective and risk factors for pain. The main aim of the present study was to investigate associations between prevalence of daily pain, self-efficacy, sleep duration, and symptoms of depression in a representative sample of Norwegian adolescents.

**Methods:**

A comprehensive cross-sectional survey was completed by 12,867 junior high school students and high school students (response rate: 90%) aged 14–19 years. Logistic regression models were adjusted for age, gender, and parental educational level.

**Results:**

We found a high prevalence of daily pain among adolescents, especially among girls (19%) compared with boys (7%). Short sleep duration was associated with increased odds ratios (ORs) of pain in the shoulders/neck (OR 1.3; 95% CI 1.3–2.0) and stomach (1.7; 1.2–2.4). Symptoms of depression were associated with increased ORs for all measured types of daily pain, including head (3.7; 3.0–4.6), shoulders/neck (3.9; 3.1–4.8), joints/muscles (4.3; 3.3–5.6), and stomach (5.5; 4.1–7.4). By contrast, self-efficacy was not associated with any form of daily pain.

**Conclusion:**

Given the burden of pain, high incidence of pain problems, and strong association between pain and depression and, to some degree, short sleep duration, co-occurring symptoms may be an important area for research in the public health field. The results highlight the importance of early identification and prevention. Longitudinal studies are needed to understand better pain problems and their underlying mechanisms with the aim of developing targeted interventions.

## Introduction

Studies have estimated that 20 to 35% of children and adolescents worldwide are affected by pain [1–5]. A systematic review reported that up to 25% of adolescents experience pain for several days each week [6]. Variations in the prevalence of pain among adolescents have been reported, but most population-based studies have identified a trend toward a high and increasing prevalence in the past decade, especially in adolescent girls [2, 4, 7, 8]. The most commonly reported types of pain are headache, abdominal pain, backache, and limb pain, and many children report pain in multiple sites [5, 9, 10]. It has also been shown that more girls than boys report pain and that living with pain affects girls more negatively than boys [3–5].

Pain is known to affect all aspects of health-related quality of life, such as physical, emotional, and social functions [11], and pain may be associated with problems with sleeping, being socially active, depression, poor school performance, and school absence [9, 11–13].

Pain may pose a problem for young adolescents during their daily activities. One study conducted in Norway found that pain and psychosocial problems in adolescence were important predictors for later health and social problems [14]. In the Norwegian population, results from the Global burden of diseases report, showed that pain is the largest single cause of disease burden, measured as disability-adjusted life years [15]. Backache and other musculoskeletal complaints frequently result in the need for sickness certifications and disability pensions in adults and young adults, which has economic and social consequences for the individual, families, and society in general [16]. The high and increasing prevalence and the serious long-term consequences of pain problems necessitate a broad understanding of the possible protective and risk factors for pain in adolescents [17].

The concept of self-efficacy (SE) is a central tenet of Bandura’s social cognitive theory [18]. SE refers to a person’s beliefs about his or her personal capacity to cope with specific challenges [19]. In pain research, SE has been defined in terms of a person’s confidence in his or her ability to cope with the symptoms, stresses, or limitations associated with a pain condition [20]. Previous studies of pain and SE have shown that a high level of SE is related to both a greater ability to cope with pain and better quality of life [21, 22].

Although previous research has considered the association between pain and SE, no studies have evaluated the relationships between SE, pain, medication use, depression, and sleep duration in a nonclinical population of adolescents. Because co-occurring symptoms of these conditions can begin early in life and persist into adulthood, it is important to gain a better understanding of the relationships between these factors. Investigating such associations could inform practice and policy. Thus, in the present study, we explored the prevalence of different types of daily pain and the associations between pain, SE, sleep duration, and symptoms of depression among a representative sample of Norwegian adolescents.

## Methods

### Design and participants

Ungdata (Young data) is a national data collection scheme designed to conduct surveys of adolescents between the ages of 14 and 19 years at the municipality level in Norway (for more information on Ungdata, (see www.ungdata.no). The present study included 12,867 junior high school students (grades 8–10) and high school students (grade 11) from 30 municipalities and 70 schools in southern Norway. In total, 15,651 students were invited to participate in the study, 11,042 junior high school students and 4609 high school students, and 12,867 students agreed to participate. The participation rates were 90% among the junior high school students and 80% among the high school students. All participants allocated 30–45 min during school hours to complete an online questionnaire. The questionnaire used in this study consisted of approximately 150 questions that were similar across all the municipalities. At least one member of the project group was present to answer any questions. Those who refused to participate were given other schoolwork by the teacher.

Legal responsibility for the Young Data survey is held by the NOVA research center of Norwegian Social Research, OsloMet.

### Ethical considerations

The study was conducted in accordance with the Declaration of Helsinki. Before participating in the study, all students were asked to provide informed consent. The parents received oral and written information about the study and were given the opportunity to withdraw their children from participation. The information letter was approved by The Norwegian Centre for Research Data (NSD). All data were collected anonymously and then analyzed by independent researchers who did not participate in the collection of the data. According to the Regional Committee for Medical Research Ethics, studies that ensure the full anonymity of all participants do not require ethical approval.

### Measures

The questionnaire included questions about sociodemographic factors, living conditions, lifestyle habits, and health status. All measures were based on adolescent self-reports.

### Outcome variables

The adolescents answered four questions about whether they had experienced pain during the previous month. In particular, they were asked whether they had experienced headache, abdominal pain, musculoskeletal pain, or neck/shoulder pain. The adolescents selected an answer from the following options: “never,” “sometimes,” “often,” or “daily.” We dichotomized the response alternatives into the categories every day and less than every day. This pain questionnaire is used in several waves of the Young data survey [23].

### Explanatory variables

SE was measured using the General Self-efficacy Scale (GSE), a 10-item psychometric scale designed to measure the optimistic self-belief in the ability to cope with a variety of problematic life demands [22, 24, 25]. The scale was developed to assess the general sense of perceived SE and aims to predict the abilities to cope with daily demands and to adapt after experiencing stressful life events. A revised five-item version of this questionnaire was used in the present study [25]. The scale was designed for the general population aged 12 years and older.

A typical item in the questionnaire is, “I always manage to solve difficult problems if I try hard enough.” The instrument uses a four-point answer scale ranging from 1 = “completely wrong” to 4 = “completely right.” The total score on the GSE ranges from 0 to 100. A higher score denotes a higher level of SE. This scale has been shown to be reliable and valid, as indicated by a Cronbach’s α between 0.75 and 0.90 [22, 25, 26].

Sleep duration was measured as a continuous variable by asking the respondents how many hours they usually sleep on a typical weekday. The responses were then dichotomized into the categories < 8 h (short sleep duration; reference category) and ≥ 8 h [27].

Depressive symptoms were measured using a six-item scale, which is based on the Hopkins Symptom Checklist-90 [28, 29]. The adolescents were asked whether, during the past week, they have been affected by any of the following: 1) “felt that everything is a struggle;” 2) “had sleep problems;” 3) “felt unhappy, sad, or depressed;” 4) “felt hopelessness about the future;” 5) “felt stiff or tense;” or 6) “worried too much about things.” Each item was rated based on four response alternatives: 1 = “not been affected at all;” 2 = “not been affected much;” 3 = “been affected quite a lot;” and 4 = “been affected a great deal.” The mean scores were computed, and the results were dichotomized using a cutoff value of ≥3.0 to classify adolescents as 1 = with depressive symptoms versus 0 = with no depressive symptoms. This cutoff value has been used in Norway in previous population studies [23], and the depressive scale has been psychometrically evaluated among Norwegian adolescents and shown to have good reliability (person separation index 0.802) [30].

### Control variables

The grade level was applied as a proxy for the participants’ age and was measured by asking the respondents for their school grade level. Grade level was included as a continuous variable in the multivariate analyses.

Gender was assessed by asking the respondents whether they were male or female (reference category).

Parental socioeconomic status was assessed by asking the respondents whether their mother and/or father had a college/university education. The response categories were binary comprising “no” or “yes” (high educational level as the reference category) for both the maternal and paternal educational levels.

The use of pain medication was assessed by asking respondents how often they had used nonprescription drugs (e.g., acetaminophen or ibuprofen) during the past month. The question had five response alternatives: “never;” “less than once a week;” “once a week;” “more than once a week;” and “every day.” These response alternatives were dichotomized into the categories of every day (high medication use; reference category) and less than every day.

### Statistical analysis

All statistical analyses were performed using IBM SPSS Statistics (version 24.0; IBM Corp., Armonk, NY, USA). For the data presented in Table 1, the χ^2^ test was used to identify gender differences in pain frequency, SE, sleep duration, symptoms of depression, age, parental educational level, and medication use. Multiple logistic regression was used to explore the relationships between daily pain incidence (head, shoulders/neck, joints/muscles, stomach, and total daily pain) and SE, short sleep duration, and symptoms of depression among adolescents. Age, gender, parental educational level, and medication use were included as control variables in all models. Odds ratios (ORs) were calculated with their 95% confidence intervals (CIs), and a two-tailed *p* < 0.05 was considered to be significant.

**Table 1:** Characteristics of the participants: daily pain, age, grade, gender, parental educational level, sleep duration, use of medication, and self-efficacy (*n* = 12,867)

	Girls	Boys	p-value*
Total number of participants, n (%)	6318 (49)	6549 (51)	
**Daily pain**			
Head, n (%)	659 (11)	161 (3)	< 0.001
Shoulders/neck, n (%)	545 (9)	214 (4)	< 0.001
Joints/muscles, n (%)	312 (5)	195 (3)	< 0.001
Stomach, n (%)	287 (5)	109 (2)	< 0.001
Any of the above, n (%)	1126 (19)	438 (7)	< 0.001
**Self-efficacy**			
Self-efficacy, mean (95% CI)	60 (59–61)	70 (70–71)	< 0.001
**Sleep**			
Short sleep duration, weekdays (< 8 h), n (%)	3104 (59)	2726 (52)	< 0.001
**Depression**			
Symptoms of depression (> 3), n%	1275 (21)	366 (6)	< 0.001
**Age**			
8th grade junior high school (14–15 yrs), n (%)	1537 (25)	1523 (24)	
9th grade junior high school (15–16 yrs), n (%)	1473 (24)	1562 (25)	
10th grade junior high school (16–17 yrs), n (%)	1506 (24)	1637 (26)	
11th grade senior high school (17–18 yrs), n (%)	1649 (27)	1628 (26)	0.137
**Parental education**			
Low paternal education, n (%)	1888 (34)	1937 (34)	0.601
Low maternal education, n (%)	1661 (29)	1638 (28)	0.306
**Medication use**			
High use (more than once a week), n (%)	1420 (23)	673 (11)	< 0.001

*The chi-square test was used for categorical variables, and the independent-sample *t* test was used for continuous variables

## Results

The characteristics of the participants are presented in Table 1. The sample comprised 6549 boys (51%) and 6318 girls (49%). Compared with boys, girls reported a higher prevalence of all measured types of daily pain, including daily pain in the head, shoulders/neck, joints/muscles, stomach, or any of these locations (*p* < 0.001 for all). Boys reported higher levels of SE than girls (*p* < 0.001). More girls than boys reported short weekday sleep duration (< 8 h), symptoms of depression, and high use of medication (more than once a week) (*p* < 0.001 for both). No gender differences in age or parental educational level were identified.

Table 2 provides information about the prevalence of pain among adolescents relative to SE, sleep duration, and symptoms of depression. All of the models were adjusted for age, gender, paternal and maternal educational levels, and pain medication use. High SE was not associated with daily pain. Short sleep duration was associated with increased ORs for daily shoulder and neck pain (OR 1.3; 95% CI 1.3–2.0), daily stomach pain (1.7; 1.2–2.4), and a combination of pain measures (one or more of daily headache, shoulder and neck pain, joint and muscle pain, or stomach pain) (1.4; 1.2–1.6). The presence of symptoms of depression was associated with increased ORs for daily headache (3.7; 3.0–4.6), shoulder and neck pain (3.9; 3.1–4.8), joint and muscle pain (4.3; 3.3–5.6), stomach pain (5.5; 4.1–7.4), and pain in one or more location’ (3.9; 3.3–4.6).

**Table 2:** Adjusted odds ratios (ORs) and 95% confidence intervals (CIs) for daily pain in relation to self-efficacy, short sleep duration, and symptoms of depression (*n* = 12,867)

		Head OR (95% CI)	Shoulders/neck OR (95% CI)	Joints/muscles OR (95% CI)	Stomach OR (95% CI)	Total daily pain OR (95% CI)
Explanatory variables	High self-efficacy	1.0 (1.0–1.0)	1.0 (1.0–1.0)	1.0 (1.0–1.0)	1.0 (1.0–1.0)	1.0 (1.0–1.0)
	Short sleep duration	1.2 (1.0–1.6)	1.3 (1.3–2.0)*	1.1 (0.9–1.5)	1.7 (1.2–2.4)**	1.4 (1.2–1.6)***
	Symptoms of depression	3.7 (3.0–4.6)***	3.9 (3.1–4.8)***	4.3 (3.3–5.6)***	5.5 (4.1–7.4)***	3.9 (3.3–4.6)***
Control variables	Age	1.1 (1.0–1.2)	1.1 (1.0–1.2)	1.0 (0.9–1.1)	0.9 (0.8–1.1)	1.0 (1.0–1.1)
	Gender: girls	2.9 (2.3–3.7)***	2.0 (1.6–2.4)***	1.1 (0.8–1.4)	1.5 (1.1–2.0)*	2.0 (1.7–2.4)***
	Low maternal education	0.7 (0.6–0.9)*	1.0 (0.8–1.2)	0.9 (0.7–1.2)	0.9 (0.6–1.2)	0.9 (0.7–1.0)
	Low paternal education	1.0 (0.8–1.3)	1.1 (0.9–1.4)	1.0 (0.8–1.3)	1.1 (0.8–1.5)	1.0 (0.9–1.2)
	High medication use	6.5 (5.4–8.0)***	2.8 (2.3–3.4)***	3.0 (2.3–3.8)***	3.2 (2.4–4.2)***	4.3 (3.7–5.0)***

^*^p < 0.05

^**^p < 0.01

^***^p < 0.001

Logistic regression models were adjusted for age, gender, maternal and paternal educational levels, and use of medication. Age was associated with increased ORs for daily pain. Being female was associated with increased ORs for daily headache (2.9; 2.3–3.7), daily shoulder and neck pain (2.0; 1.6–2.4), daily stomach pain (1.5; 1.1–2.0), and a combination of pain measures (2.0; 1.7–2.4). Low maternal educational level was associated with a reduced OR for daily headache (0.7; 0.6–0.9), but parental educational level was not associated with any other measures of daily pain. Finally, high medication use was associated with increased ORs for daily headache (6.5; 5.4–8.0), shoulder and neck pain (2.8; 2.3–3.4), joint and muscle pain (3.0; 2.3–3.8), stomach pain (3.2; 2.4–4.2), and a combination of pain measures (4.3; 3.7–5.0).

## Discussion

In the present comprehensive study, which included more than 12,000 adolescents, we examined the prevalence of daily pain, SE, sleep duration and symptoms of depression. Our findings demonstrated a high prevalence of daily pain among adolescents, especially among girls. Moreover, pain problems covaried with short sleep duration and symptoms of depression, however our analysis did not confirm any association between high SE and daily pain.

The high prevalence of daily pain is in line with previous studies. A study noted that 19.8% of Danish adolescents report daily pain, with a higher prevalence among girls than boys. In the age group 12–19 years, 13% of the boys and 23% of the girls reported daily pain [8]. Our results indicated a higher prevalence of daily pain in all of the investigated locations, including the head, shoulders/neck, joints/muscles, or stomach, in girls than in boys. This finding is consistent with results of the Norwegian Young-HUNT study, which found that 44% of the adolescents experienced pain at least once a week during the preceding 3 months and that the prevalence was higher among girls than boys and increased with age for all pain types [31, 32]. Despite differences in the prevalence of pain between studies, most studies conducted in nonclinical populations have detected a trend toward a high and increasing prevalence of pain among adolescents over the past decade, especially among adolescent girls [2, 10, 33].

In view of the significant gender differences in pain prevalence, researchers have suggested that gender may be an explanatory factor for pain [2, 3, 14, 34]. It has been suggested that gender differences in pain prevalence may be explained by the interaction of different factors such as; biological factors, including hormones, psychological factors, such as anxiety and negative affect, and sociocultural factors, including gender role expectations of pain [35].

Our findings emphasize the vulnerability of adolescent girls. The high prevalence of pain in adolescent girls is a cause of concern. In our study, pain in adolescents was associated with widespread negative effects on psychological health. Consistent with previous research, our study found relationships between pain and depression. Previous studies of pain have shown that pain, especially headache, is associated with anxiety and depression [36, 37]. Symptoms of depression were associated with increased ORs of daily headache, shoulder and neck pain, muscle pain, stomach pain, and a combination of pain measures in our study. Our results also show that pain problems covary with sleep problems. We found that short sleep duration and high pain medication use were associated with increased ORs of experiencing some form of daily pain. These results are consistent with those of a study of Swedish adolescents, which reported an association between short sleep duration and increased odds of pain problems [38]. Other studies have also demonstrated a link between poor sleep quality and increased pain during adolescence [39, 40]. Disturbed sleep may affect the lives of adolescents in various ways and may manifest as issues associated with school-related problems, poor concentration, and social problems [16]. Thus, sleep may play a significant role in the adolescent experience and management of pain. Early conceptual models have described a bidirectional relationship between pain and sleep, in which pain can cause sleep problems, and disturbed sleep can lead to the development of pain [41].

SE is a positive phenomenon, and it is particularly relevant from a health promotion perspective because SE focuses on resources rather than problems [24, 26, 42]. In pain research, studies have shown that a high level of SE may be characterized as a protective psychological resource, and related to several positive health outcomes, such as greater ability to cope with pain, better quality of life, and fewer somatic symptoms [22, 43, 44]. Surprisingly, our analyses did not reveal a significant association between high SE and daily pain in any location of any form. One possible explanation is that we measured daily pain, whereas earlier studies of pain and SE focused mainly on persistent pain [22, 43].

Results from our study show that at attention should be paid to adolescents with pain in the public health work.. Early adolescence is a critical period for the emergence of pain. Children with pain are at risk for growing up to become adults with pain and mental health comorbidities [45]. Given the high incidence of pain problems in adolescence reported in the past decade, especially in girls, the association with psychological problems and short sleep duration, a broad biopsychosocial approach is important to understanding pain in adolescents.

### Strengths and limitations

The strengths of our study are the large sample size, high response rate, and use of well-validated questionnaires. Our study also has some limitations. In particular, substantial causal inferences cannot be made, and directions of the observed relationships cannot be determined because of the cross-sectional design. All of the instruments used were self-reportedwith the risk of information bias and were dependent on both the adolescents’ memory and judgment. Moreover, adolescents who were absent from school on the day of the study did not participate, and we could not assess whether the participants and nonparticipants differed in terms of the variables considered. In addition, we measured daily pain during the previous month, but we obtained no information about pain duration or chronic or persistent pain, which means that reported pain may include both long-term and short-term pain.

## Conclusion

Overall, our results demonstrate that daily pain is common, especially among adolescent girls. We found a higher prevalence of all types of daily pain in girls than in boys and that pain problems covaried with short sleep duration and symptoms of depression. Adolescent boys reported higher levels of SE than adolescent girls. However, the present study did not confirm an association between high SE and the prevalence of daily pain among adolescents. Given the burden of pain, the high incidence of pain problems in adolescence, and the strong association with depression and, to some degree, short sleep duration, co-occurring symptoms should be considered an important area in public health work. The results highlight the importance of early identification and prevention. Longitudinal studies are needed to understand better pain problems and their underlying mechanisms with the aim of developing targeted interventions.

## Data Availability

The data that support the findings of this study are available from the corresponding author upon reasonable request.
